# Artificial intelligence and image processing framework for automated airway invasion detection and residue classification from swallowing endoscopy

**DOI:** 10.1038/s41598-026-44495-4

**Published:** 2026-04-10

**Authors:** Luiza Araújo, Enzo Rangel, Anibal Cotrina-Atencio, Vitória Gomes dos Santos, Ana Maria da Costa dos Santos Reis, Hipólito Magalhães, Lidiane Ferreira, André Felipe Oliveira de Azevedo Dantas, Caroline Cunha do Espírito-Santo

**Affiliations:** 1 Postgraduate Program in Neuroengineering, Edmond and Lily Safra International Institute of Neuroscience, Santos Dumont Institute, Zona Rural, Macaíba, 59288-899 RN Brazil; 2 Department of Speech-Language Pathology, Associated Graduate Program in Speech-Language Pathology – UFPB/UFRN/UNCISAL, Natal, 59012-570 Brazil; 3 Graduate Program in Electrical and Computer Engineering, Federal University of Rio Grande do Norte – UFRN, Natal, 59078-970 Brazil; 4 Department of Computing and Electronics, Federal University of Espírito Santo – UFES, Vitória, 29075-910 Brazil; 5 Department of Biomedical Engineering, Federal University of Rio Grande do Norte – UFRN, Natal, 59078-970 Brazil; 6 Multiprofessional Residency Program in Health Care for People with Disabilities, Santos Dumont Institute - SDI, Macaíba, 59280-000 Brazil; 7 Department of Surgery, Federal University of Rio Grande do Norte – UFRN, Natal, 59012-300 Brazil

**Keywords:** Deglutition disorders, Endoscopy, Pharyngeal residues, Respiratory aspiration, Neural networks, Computer, Diseases, Engineering, Health care, Medical research

## Abstract

Fiberoptic endoscopic evaluation of swallowing (FEES) is a widely used instrumental method for dysphagia, but its interpretation depends on lighting and evaluator experience. This study developed and validated a framework artificial intelligence (AI) and image processing to detect penetration, aspiration, and classify residues according to the Yale Pharyngeal Residue Severity Rating Scale (YPR-SRS). The system combines anatomical tracking of the epiglottis, arytenoids, and vocal folds with contrast adjustments, color-based segmentation, and enhancement filters, optimizing the visibility of clinical events. In validation with 60 videos, accuracy was of 0.90 for penetration and 0.87 for aspiration, with sensitivities of 1.00 and 0.90, specificities of 0.80 and 0.85, and Kappa values of 0.80 and 0.75. For residues in the pyriform sinuses, accuracy ranged from 0.91 to 0.95, sensitivity from 0.91 to 1.00, specificity from 0.83 to 1.00, and Kappa = 0.87. In the valleculae, accuracy ranged from 0.95 to 1.00, with sensitivity and specificity of 0.91–1.00 and Kappa = 0.91. These findings highlight its assistive potential for FEES analysis and clinical decision-making support in penetration, aspiration, and pharyngeal residue classification, a capability not previously reported.

## Introduction

The oropharyngeal dysphagia is characterized by sensorimotor and functional impairments affecting the coordinated activity of the stomatognathic system during swallowing which may result from neurological, structural, functional, or sensorimotor alterations^1^. It is a common condition associated with neurological impairments, with a prevalence of approximately 67% in cases of stroke and over 70% in cases of Parkinson’s disease (PD) [2,3].

This disorder disrupts the coordinated oropharyngeal transition and airway protection mechanisms during swallowing, which can result in the accumulation of food residues^1–3^. This residue accumulation can occur, either in isolation or simultaneously, in the valleculae and/or pyriform sinuses. The quantity of residue in these regions is associated with an increased risk of laryngotracheal penetration and aspiration, which are the main complications reported in this clinical condition^4^.

The fiberoptic endoscopic evaluation of swallowing (FEES) is one reference standards instrumental assessments that complements clinical evaluations of dysphagia by directly visualizing swallowing, pharyngeal residues, and penetration and aspiration events. It provides an axial view of the laryngopharyngeal surface and surpasses the Videofluoroscopic Swallowing Study (VFSS), in detecting penetration and pharyngeal residues^5,6^. In FEES, penetration and aspiration are defined by the presence of material above and below the level of the vocal folds, respectively, whereas pharyngeal residue is typically classified using the ordinal criteria of the Yale Pharyngeal Residue Severity Rating Scale (YPR-SRS), one of the available visuoperceptual methods for defining residue, based on presence in the valleculae and/or pyriform sinuses^7^.

Both the detection of penetration and aspiration and the application of the YPR-SRS rely on visual examiner interpretation and are therefore susceptible to bias^7^. The YPR-SRS is based on an anatomically defined ordinal classification focused on residue location and quantity, without directly incorporating broader functional aspects of swallowing that are clinically relevant, which represents an important limitation for its interpretation in clinical decision-making^7^. Nevertheless, it remains widely used in clinical settings, and ensuring its consistent and objective application continues to be a central challenge in routine patient care.

Previous studies have shown that less experienced clinicians achieve accuracy rates below 60% in detecting penetration and aspiration during FEES^8^. This is likely due to the lack of well-defined objective criteria for detection in this examination, which, when combined with the extrapolation of parameters used in VFSS, contributes to imprecise interpretation of the results.

In the case of the YPR-SRS, although the scale demonstrates good inter- and intra-rater reliability in assessing the location and amount of pharyngeal residue^7,9–11^, its performance has been primarily established under controlled experimental conditions. The consistent findings reported in the literature are largely derived from trained specialists evaluating pre-selected FEES images, often with prior knowledge of the target anatomical region^7,10,11^. While such methodological standardization is essential for instrument validation, routine clinical practice introduces additional sources of variability, including anatomical diversity and equipment-related lighting differences^8^, which may affect the consistency of visual interpretation. Within FEES, residue classification using the YPR-SRS complements penetration and aspiration outcomes. Together, these measures provide information on swallowing efficiency and safety, respectively, which highlights the need for more objective and reproducible interpretations.

Given these limitations, several strategies regarding to the enhancement of the image quality or the automatic detection have been proposed to improve diagnostic accuracy. For example, the adoption of high-definition equipment with enhanced color contrast has facilitated the visualization of relevant events^12^. However, in addition to high financial costs and the need for specialized training, these technologies do not address the core issue: variability in examiner interpretation^13^. In this context, artificial neural networks based on artificial intelligence (AI) techniques are being proposed for the automatic detection of laryngeal structures, penetration/aspiration, and residues^13,14^. Despite their promise, these approaches involve high computational costs, do not correct by itself illumination distortions present in the exam recordings, and consequently do not classify efficiently residue severity according to the YPR-SRS scale, thus maintaining diagnostic imprecision^13^.

In this sense, these approaches remain fully dependent on the quality of the original exam recording, particularly for the identification of residues in the valleculae and pyriform sinuses, where evaluations are often impaired by low image quality, making it difficult to distinguish between severity levels^13–16^. In addition, the application of the YPR-SRS considers each swallow in isolation; therefore, in cases of successive swallows or bolus segmentation, the assigned score may vary between swallows depending on the specific time point evaluated^7^. These limitations can be partially addressed through image processing techniques such as image sharpening or object segmentation; however, such approaches remain conditioned by the position of the camera relative to the anatomical structures^14,16,17^. In this context, neural network–based tools for anatomical landmark tracking, such as DeepLabCut (DLC), have been explored as a complementary strategy for the analysis of laryngeal movement and may assist in the temporal selection of critical moments, thereby supporting more consistent identification of residues in anatomically relevant regions, even in the presence of anatomical and spatial variability^16,17^.

Building on this, the present study proposes the integration of anatomical tracking and color-based segmentation in FEES examinations. To this end, the study combines dynamic tracking of laryngeal structures using DLC with color segmentation, automatically highlighting residues through the detection of dyed food during the exam. By concentrating computational costs solely in the training phase, this approach allows for lightweight and efficient application, with potential to standardize diagnostics. Accordingly, the objective of this study was to develop and validate an AI- and image processing–based framework for the classification of pharyngeal residues according to the YPR-SRS, as well as for the identification of penetration and aspiration in FEES.

## Methods

### Database information

This study analyzed 112 FEES exams from a private database of the HUOL dysphagia outpatient clinic, collected between January 2020 and September 2024. Of these, 52 videos were used to train the convolutional neural network for anatomical landmark detection, whereas 60 previously unseen videos were reserved for evaluating the performance of the complete framework.

The videos were selected to ensure that each dataset contained similar proportions of absence and presence of penetration and aspiration, as well as pharyngeal residues categorized as absent, trace, mild, moderate, or severe, thereby providing a representative distribution of swallowing characteristics across normal and impaired patterns.

All exams in this database included complete diagnostic reports previously established as part of routine clinical practice and retrieved retrospectively. These reports were based on pre-existing consensus assessments between an otolaryngologist and two speech-language pathologists, all with more than 10 years of experience in FEES and specialized training in dysphagia. The assessments were performed jointly by the three professionals during the FEES examination, as were the final diagnostic decisions regarding the presence of penetration and aspiration, residue severity, and dysphagia severity. No experts were prospectively recruited for the purposes of this study, as their assessments had been performed prior to and independently of the present research.

These expert consensus reports described residue severity patterns, all rated according to the standardized and internationally validated YPR-SRS scale^7^. Penetration and aspiration events were recorded as either present or absent. Dysphagia severity was defined based on a clinical synthesis of swallowing efficiency and airway safety observed during FEES. Absence of dysphagia was characterized by efficient bolus clearance without pharyngeal residue or airway invasion. Mild dysphagia was defined by minimal pharyngeal residue with preserved airway protection. Moderate dysphagia was characterized by moderate pharyngeal residue and or laryngeal penetration without aspiration. Severe dysphagia was defined by severe pharyngeal residue and or the presence of aspiration associated with marked impairment of swallowing efficiency. In cases of airway invasion accompanied by spontaneous cough or effective airway clearance, expert raters based severity classification on the final airway status after the protective response and the amount of residual pharyngeal material, rather than on the initial invasion event alone.

All procedures were performed using a flexible nasofibroscope with a 3.2 mm diameter, from either Olympus or Pentax. The videos had a sampling rate of 30 frames per second (fps) and an average duration of approximately 7 min.

Inclusion criteria for the study comprised videos of individuals with (1) neurogenic oropharyngeal dysphagia (OD) and (2) without OD (normal). During the FEES procedure, boluses of different consistencies were offered at IDDSI levels 0, 2, 4, and 7, in accordance with the International Dysphagia Diet Standardisation Initiative (IDDSI)^18^, in order to promote variability in bolus consistency. Individuals without penetration, aspiration, or pharyngeal residue were included to allow evaluation of whether the algorithm could correctly identify the presence or absence of these events in different situations.

For this study, we excluded videos with incomplete oropharyngeal exams, blurred images, or poor visibility of oropharyngeal structures due to technical issues, especially those in which the key points reached less than 70% accuracy in more than 50% of the frames after anatomical tracking.

### Framework based on AI and image processing for swallowing event detection

#### Transfer learning–based oropharyngeal anatomical tracking using Resnet50

For the automatic analysis of FEES videos, the system must accurately identify the anatomical structures involved in swallowing, since the detection of pharyngeal residue, penetration, and aspiration events depends on the correct localization of the regions where these events occur. To enable this recognition, an AI-based approach was developed using data manually annotated by another group of clinical professionals with experience in dysphagia and FEES, independent from those involved in the diagnostic consensus.

A convolutional neural network based on ResNet-50 with transfer learning (implemented using the open-source DLC software) was employed to automatically identify and return bidimensional coordinates of three laryngopharyngeal structures, the epiglottis, arytenoids, and vocal folds, located near the valleculae, pyriform sinuses, and glottic region, respectively associated with residue, penetration, and aspiration. These structures were color-coded blue, green, and red (Fig. 4a).

The deep learning model was trained on 80% of the 52 available FEES videos for this purpose, while the remaining 20% were reserved as an independent validation set and not used at any stage of training.

From each video in the training set, 500 frames were automatically extracted using the OpenCV library, assisted by the K-means clustering algorithm. This approach ensured that the selected frames were sufficiently similar within clusters and distinct across clusters. These frames were manually annotated in order for the network to learn to accurately identify the locations of the target structures. As a result, the neural network generates a video with the structures automatically marked, accompanied by a comma-separated values (CSV) file containing the horizontal and vertical coordinates of these structures, as well as the accuracy of each marked point in every frame.

The model performance for this annotation task was evaluated by computing the mean absolute error (MAE), which reached 5.2 pixels. Errors of this magnitude are generally considered low in pose estimation tasks, indicating that the model effectively generalizes the localization of anatomical landmarks in previously unseen videos^19,20^.

The quantification of residue and detection of penetration–aspiration events were computed from regions of interest derived directly from these anatomical landmarks. The observed MAE defines the geometric tolerance of the entire pipeline, as it bounds the precision with which anatomical regions can be delineated and, consequently, constrains the reliability of downstream clinical classification.

#### Enhancing anatomical visibility for detection residues, aspiration and penetration

After the model demonstrated the ability to generalize the identification of anatomical regions, image processing algorithms were developed to integrate tracking into video preprocessing, including brightness and contrast adjustments. At this stage, the framework operates in a semi-automated manner: the researcher selects the events, and subsequent processing is fully automated. The software requires manual specification of time intervals corresponding to swallows of interest. Based on these intervals, short video segments are automatically extracted and split into individual frames. The researcher then selects one frame immediately before and one immediately after the whiteout event, choosing those with the highest accuracy, as indicated by the neural network.

**Fig. 1:**
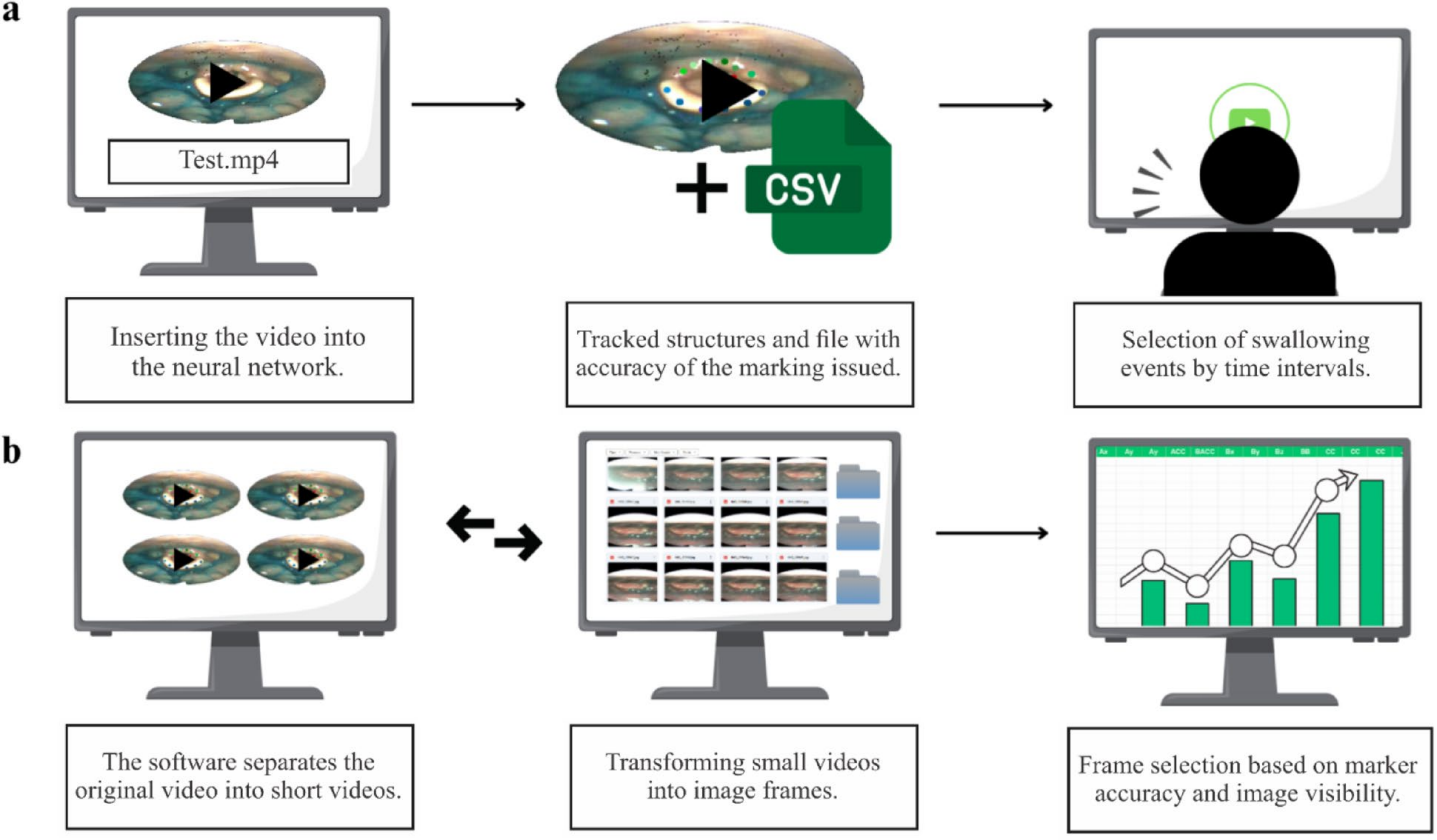
(**a**) The video is input into a neural network for automatic anatomical tracking, and the researcher selects the swallowing events of interest based on the tracked data. (**b**) Corresponding video segments are extracted and converted into static frames, which are selected for processing according to the tracking accuracy of the anatomical landmarks.

This integration, combined with anatomical markers, enhanced the visibility of anatomical structures, facilitating the identification of penetration and aspiration events in the glottis region and residue accumulation in the valleculae and piriform sinuses. Figure 1 illustrates the workflow of the system.

To enable the automatic identification of penetration, aspiration, and residue accumulation events, the software performs a sequence of image enhancement procedures^21^. First, color space conversions to LAB space (color space) and HSV space (color space) are applied to allow for targeted brightness and saturation corrections. Contrast is optimized through adaptive histogram equalization, while Gaussian (5 × 5, σ derived from kernel size) and median (3 × 3) filters are used to reduce artifacts, they smooth intensity fluctuations and suppress impulse noise, respectively, preserving edges. A controlled sharpening filter is then applied to enhance contours without increasing noise.

These adjustments are essential due to the visual similarity between pharyngeal residues and anatomical structures, which often share light tones such as white and pink. This similarity makes visual differentiation challenging, particularly when residues are minimal or lightly colored. To address this, the software enhances blue tones - associated with the dye used during examinations, thereby highlighting the regions of interest (Fig. 1b).

Next, the image is converted to grayscale and segmented using thresholding, a technique that facilitates the separation of anatomical structures from residues. Morphological operations such as hole filling, large contour filtering, erosion, closing, and opening are applied to remove background noise and connect areas fragmented by glare.

**Fig. 2:**
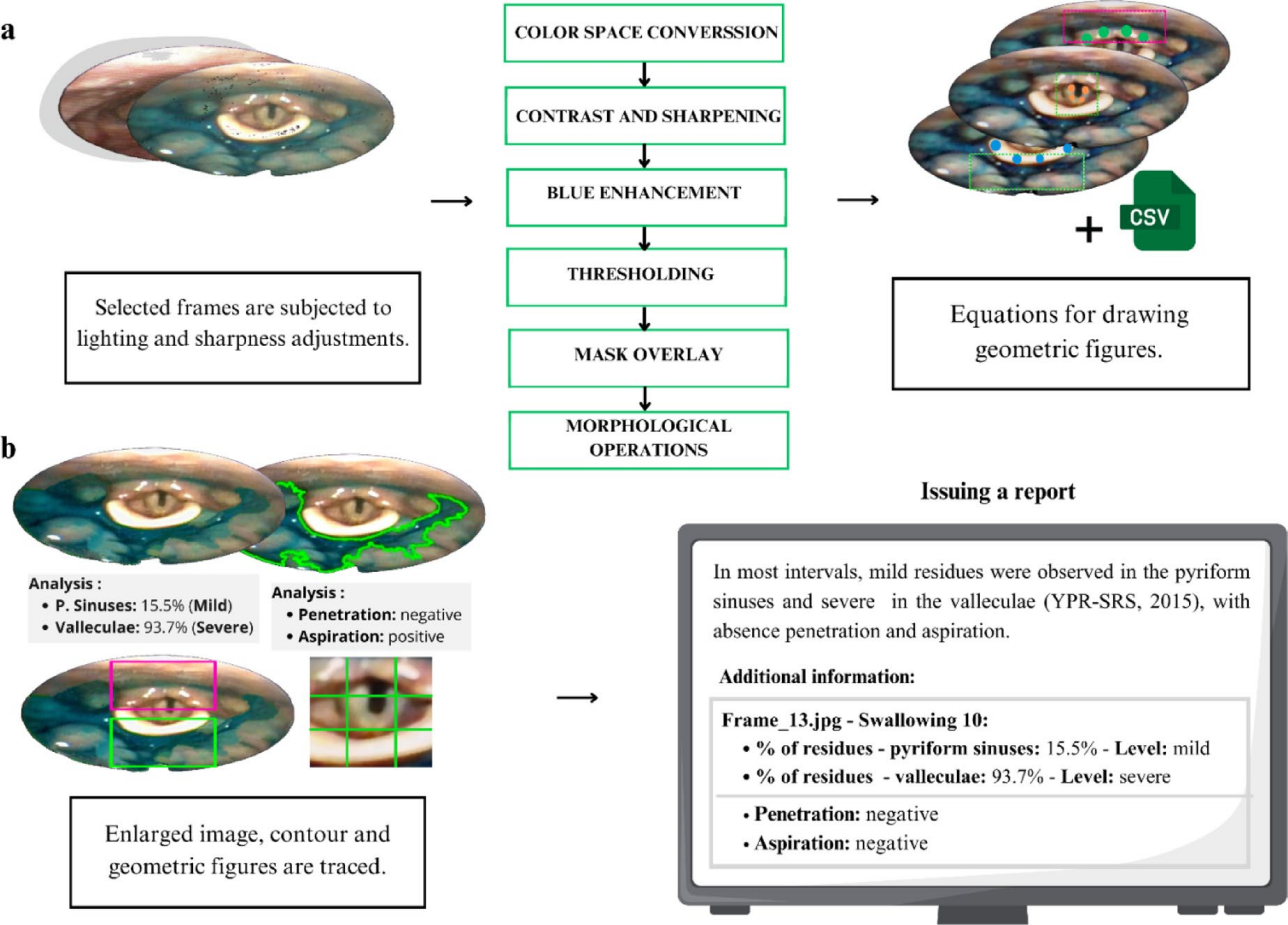
(**a**) Images selected in the neural network stage undergo a sequence of visual quality enhancement procedures. Based on the tracking information of the arytenoids, vocal folds, and epiglottis provided by the network, the pyriform sinuses, valleculae, and glottis regions are delineated using rectangular and square geometric shapes, respectively. (**b**) After delineation of the structures of interest, the algorithm outputs images with the regions of interest highlighted, according to the YPR-SRS. Pharyngeal residues are contoured, the glottis region is magnified, and a report summarizing the image-based findings is generated. This report provides an overview of swallowing events identified by the system. It specifies the swallows of interest selected by the researcher, along with their time intervals, and includes alerts regarding the severity of residues and the occurrence of penetration and/or aspiration events.

At the end of the process, the segmented mask is overlaid onto the original image and converted back to the Red, Green, and Blue (RGB) color space, preserving an interpretable visualization of the structures. Final adjustments to brightness, contrast, and sharpness are reapplied to ensure better contour definition and facilitate the identification of clinically relevant events (Fig. 2a, b).

#### Combining AI and image processing for classification of residues and identification of aspiration and penetration

**Fig. 3:**
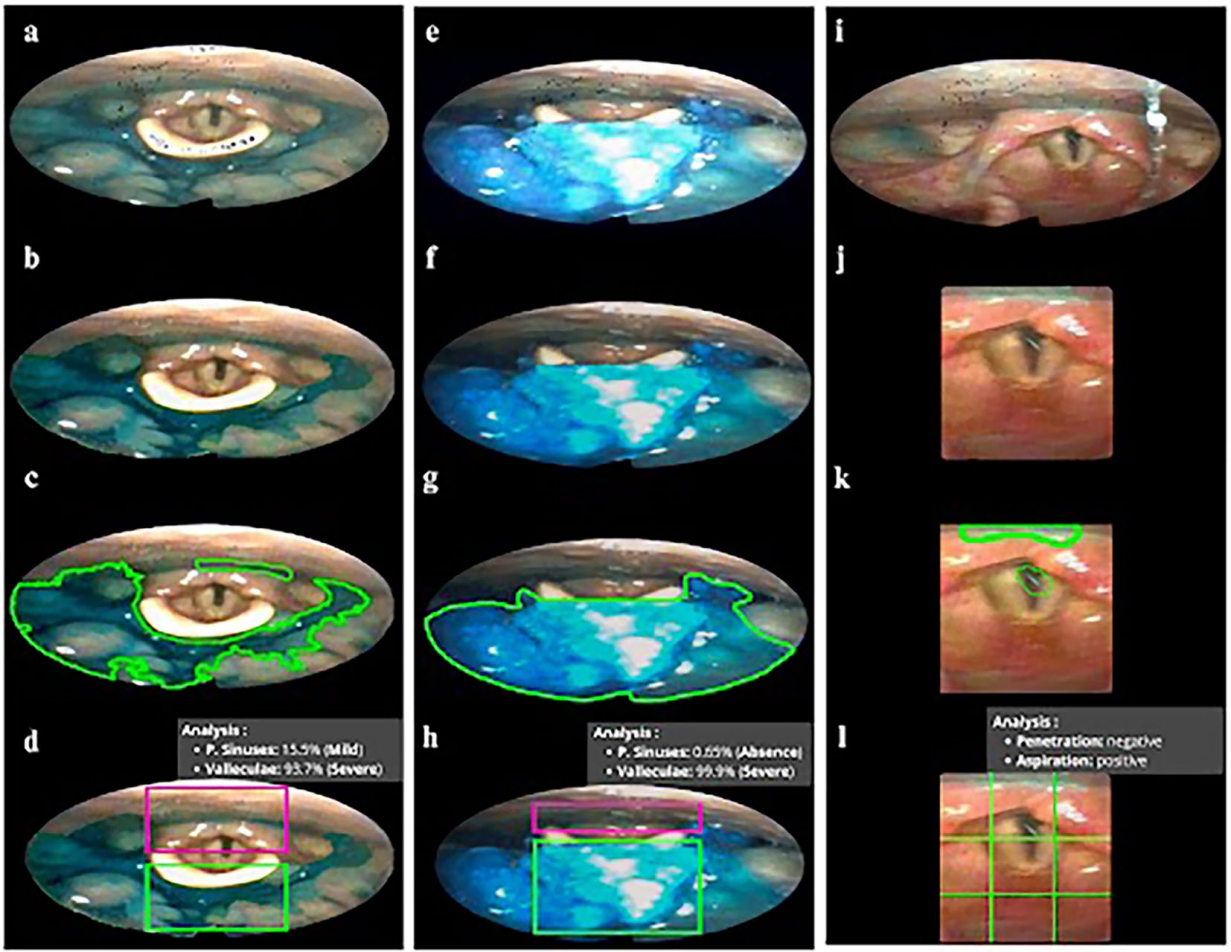
(**a**), (**e**), (**i**) = images before processing; (**b**), (**f**), (**j**) = images after lighting and sharpness adjustments; (**c**), (**g**), (**k**) = images with pharyngeal residues and aspiration events outlined; (**d**), (**h**) = image with rectangles drawn in the pyriform sinuses and valleculae, according to the anatomical limits established in the YPR-SRS scale; (**l**) = enlarged images in square format with grids in the glottis region for locating penetration and aspiration; (**d**), (**h**), (**l**) = processed images showing framework results for quantification of pharyngeal residues and detection of penetration and aspiration events.

Based on the color-adjusted images, the spatial coordinates of the anatomical structures are used to define the regions of interest. The software employs these coordinates, extracted by the neural network, to draw a square over the glottis area and magnify the region. Additionally, it delineates rectangular regions over the pyriform sinuses and the valleculae that allow the identify automatically the severity of the residues according to the YPR-SRS scale (Fig. 3d, h).

**Fig. 4:**
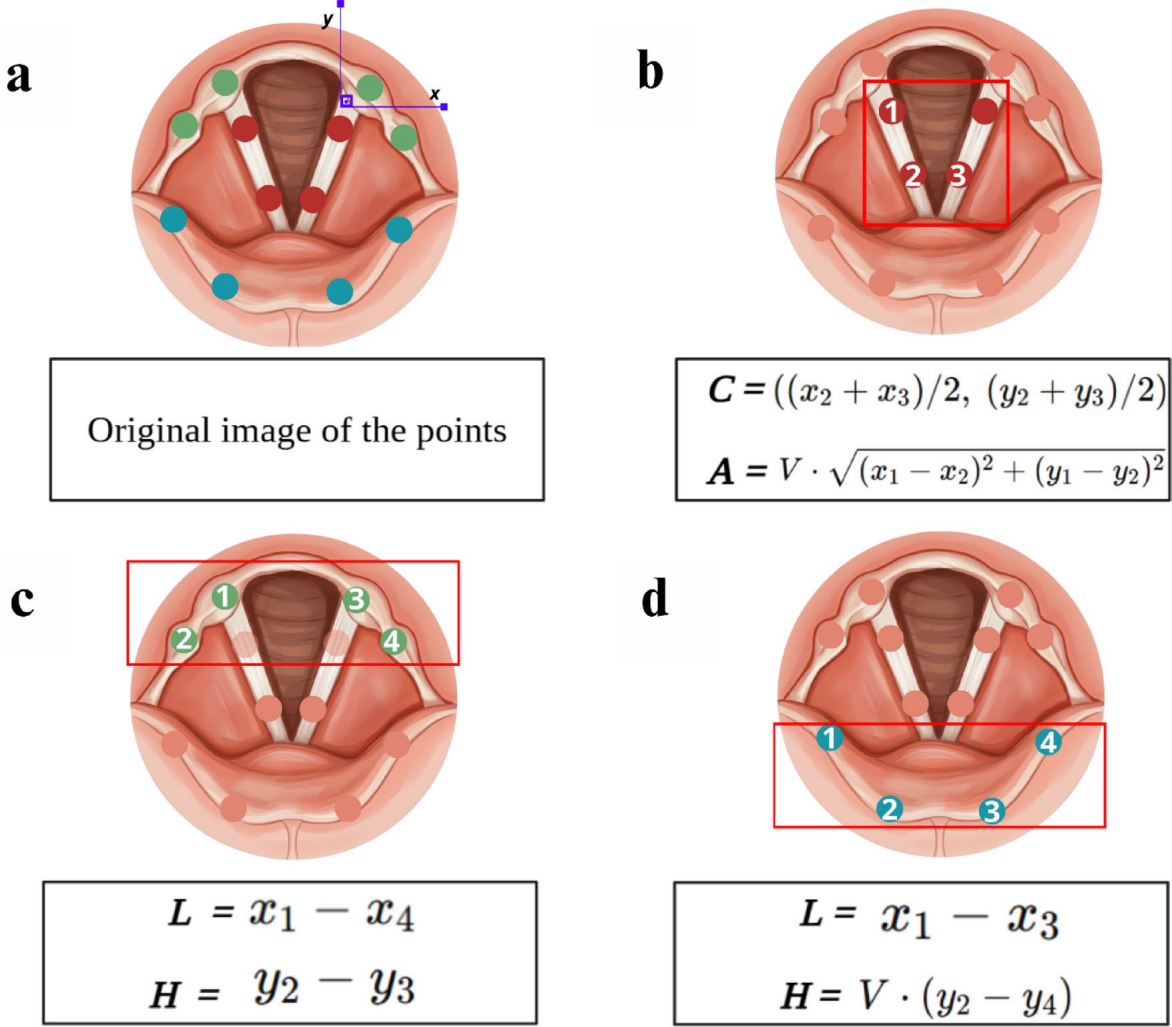
(**a**) Original image of the points marked with DLC; (**b**) Calculation of the center of the vocal folds, performed separately, from the average position of the x and y axes of points 2 and 3, which will allow the construction of a square, whose area is based on the Euclidean distance between two points, multiplied by *V* and squared; (**c**) The rectangle over the pyriform sinuses is determined from the difference between the x coordinates of points 1 and 4, to define the width, and between the y coordinates of points 2 and 3, multiplied by *V*, to define the height; (**d**) The rectangle over the valleculae is constructed from the difference between the x coordinates of points 1 and 3 for the width and the y coordinates of points 2 and 4 for the height. The calculation of the residue percentage is performed exclusively within these delimited geometric areas, considering the ratio of blue pixels relative to the total number of pixels in each outlined shape.

The regions of interest are automatically delineated by the software based on simple mathematical calculations, as illustrated in Fig. 4b–d. In the glottic region, for example, a central point is calculated by averaging the coordinates of points 2 and 3 (Fig. 4b). This point serves as a reference for creating a square whose diagonal length (A) is proportional to the distance between points 1 and 2, multiplied by a variable *V*. From this square, a 3 × 3 grid enlargement is generated to facilitate the differentiation between penetration and aspiration events.

For each swallow, the frame immediately before and after the whiteout is selected by the researcher based on the highest marker accuracy, ensuring a consistent anatomical reference, as is also done for the detection of pharyngeal residues. In the enlarged image of the glottic region, the software calculates the percentage of blue-stained pixels and identifies the quadrant with the highest concentration.

Penetration and aspiration events are then defined in a binary and deterministic manner: if residue is present in the central quadrants corresponding to the glottic entry, the algorithm flags an aspiration event; if residue is present in other quadrants, a penetration event is flagged; if residue is present in both central and peripheral quadrants, the algorithm flags both penetration and aspiration events. These binary alerts serve as a clinical support and screening tool, assisting the clinician in the diagnostic decision-making process.

For the pyriform sinuses (Fig. 4c), the width of the rectangle was determined from the difference between the x-coordinates of points 1 and 4, whereas its height was determined by the difference between the y-coordinates of points 2 and 3, multiplied by a variable V, ensuring that the region was adjusted to the anatomy. In the case of the valleculae (Fig. 4d), the width and height of the rectangle were delineated from the difference between the x-coordinates of points 1 and 3 and the difference between the y-coordinates of points 2 and 4, respectively.

The correspondence between pharyngeal residues and the YPR-SRS scale was established through pixel-based quantification within anatomically defined regions identified by neural network landmarks. The algorithm iterates over all pixels within each region, identifying those stained blue as residues and calculating their proportion relative to the total number of pixels within the geometrically defined area. Based on this proportion, the severity was automatically classified according to the YPR-SRS scale as follows: absence (< 1%), trace (1–5%), mild (5–25%), moderate (25–50%), or severe (> 50%). Residues are quantified strictly in the pyriform sinuses, using the height of the residue relative to the aryepiglottic folds as a reference, and in the valleculae, using the level of filling relative to the epiglottis as reference.  .

At the end of processing, the software outputs information regarding the quantity and severity of the residues, in addition to indicating the presence of penetration and potential aspiration events. These outputs represent a clinical interpretation in which the residue percentage is automatically categorized into severity levels based on the YPR-SRS scale. Processed images with outlined structures, enlarged views of the glottic region, and versions with and without grid overlays were also generated (Fig. 2b).

#### Validation against expert consensus reports

After this procedure, the system needed to be validated. Thus, a new sample of 60 videos (not previously used) was selected and processed by the neural network. The network automatically annotated the videos with the points shown in Fig. 1 and generated CSV files for each of them.

These videos were used to evaluate the performance of the proposed framework, which identifies penetration and aspiration events, as well as quantifies pharyngeal residue according to the YPR-SRS scale. The results produced by the system were compared with the retrospective consensus reports prepared by three professionals with over 10 years of experience in FEES, who were independent of the group responsible for the manual annotations used in the training phase, as described in the Database Information section.

As these reports were retrieved from a clinical database where assessments were performed jointly as part of routine practice, individual ratings for each professional were not available, making a separate inter-rater reliability analysis unfeasible. Thus, the consensus served as the reference standard for accuracy assessment, ensuring agreement with established expert evaluations and minimizing the likelihood of error.

The primary outcome was the performace metrics of the AI system in identifying the exact anatomical sites and amount of residue, using the YPR-SRS scale as the reference standard. Secondary outcomes included the inter-rater reliability between the automated system and the expert consensus reports for residue classification, and the performance metrics in detecting oropharyngeal penetration and aspiration events. In all cases, AI performance was compared with expert ratings derived from the consensus reports.

### Statistical analysis

A descriptive statistical analysis was conducted to characterize the demographic data, underlying medical conditions, and the severity of dysphagia in the sample. Means were used for continuous variables, while absolute and relative frequencies were applied to categorical variables. The normality of the data was assessed using the Shapiro–Wilk test, and due to non-normal distribution (*p* < 0.05), non-parametric tests were applied.

The software’s performance was evaluated based on sensitivity, specificity, accuracy, positive predictive value (PPV), negative predictive value (NPV), positive likelihood ratio (PLR), negative likelihood ratio (NLR), and the area under the curve (AUC). The association between the classifications provided by the software and by the expert report was analyzed using Fisher’s Exact Test, adopting a 5% significance level (*p* < 0.05).

Agreement between the software and the consensus report was assessed using Cohen’s Kappa coefficient for each swallowing-related outcome, with weighted Kappa applied to ordinal pharyngeal residue severity and unweighted Kappa applied to binary penetration and aspiration events. Kappa values were interpreted according to Landis and Koch (1977) as follows: poor (< 0.20), fair (0.21–0.40), moderate (0.41–0.60), substantial (0.61–0.80), and almost perfect (0.81–1.00)^22^.

All statistical analyses were performed using JASP software (version 0.19.3).

## Results

The video sample consisted of 34 (56.6%) male and 26 (43.3%) female individuals, with a mean age of 64 years. Among the analyzed videos, 24 (40.0%) were from individuals with Parkinson’s disease (PD), 17 (28.3%) with stroke, 7 (11.6%) had other neurological diagnoses, and 12 (20.0%) presented with symptoms of progressive neurological disorders and swallowing complaints but were still being investigated for a definitive diagnosis.

Regarding the severity of dysphagia, according to the consensus report, 19 videos (31.7%) were classified as mild, 12 (25.0%) as moderate, 12 (25.0%) as severe, and 17 (28.3%) showed no signs of dysphagia.

In Table 1, the results show that the software can predict penetration and aspiration with accuracies of 0.90 and 0.87, respectively. These high accuracies indicate a high proportion of correct diagnoses, demonstrating the software’s reliability in identifying these conditions. Additionally, the software was highly effective in detecting true positive cases of penetration and aspiration cases (sensitivity 1.00 and 0.90, respectively) and only 10–15% were incorrectly classified as positive (specificity penetration and aspiration, respectively). AUC analysis demonstrated the excellent discriminatory performance of the software for penetration and aspiration (0.90 and 0.87, respectively). For all performance metrics, Fisher’s Exact Test, indicated a statistically significant association (*p* < 0.001) between the software’s classifications and the consensus report (Table 1).

**Table 1:** Software performance measures for identifying penetration and aspiration, considering the consensual report as a reference.

Variables	Accuracy	AUC	Sensitivity	Specificity	Precision	VP+ VP-	RV+ RV-	Fisher’s test
Penetration - software vs. report	0.90	0.90	1.00	0.80	0.83	0.80 1.00	6 0	0.001*
Aspiration - software vs. report	0.87	0.87	0.90	0.85	0.85	0.89 0.85	8.5 0.16	0.001*

*Significant value (*p* < 0.05) – Fisher’s Exact Test. AUC = Area Under the Curve; VP + = positive predictive value; VP - = negative predictive value; RV + = positive likelihood ratio; RV- = negative likelihood ratio.

**Fig. 5:**
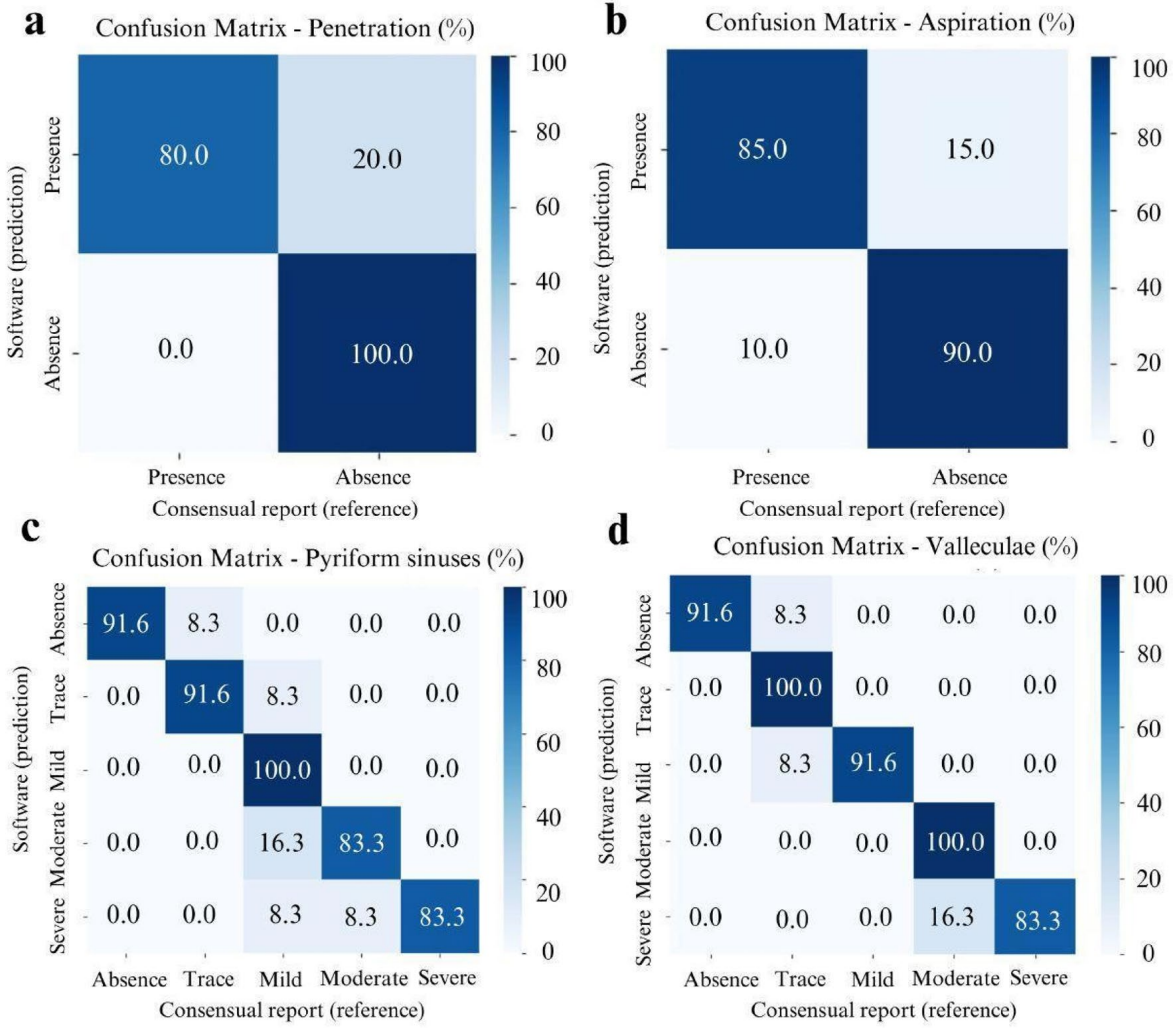
Confusion matrices comparing software predictions and expert consensus for (**a**) penetration, (**b**) aspiration, (**c**) severity classification of pharyngeal residues in the pyriform sinuses, and (**d**) severity classification of pharyngeal residues in the valleculae. Values are expressed as percentages.

The agreement between the software and the medical report, as assessed by Cohen’s Kappa coefficient, was substantial for both penetration (Kappa = 0.80) and aspiration events (Kappa = 0.75). Figure 5a, b displays the distribution of the data.

Table 2 shows the software’s performance in detecting pharyngeal residues in the pyriform sinuses across different severity comparisons. Overall, the software demonstrated high accuracy, ranging from 0.91 to 0.95, indicating a high proportion of correct classifications for both presence/absence and inter-grade residue comparisons. Sensitivity was consistently high across all analyses (≥ 0.91), reaching 1.00 in most comparisons, which indicates excellent detection of true positive residue cases. Specificity values ranged from 0.83 to 1.00, reflecting a low rate of false-positive classifications.

Precision values remained high (0.85–1.00), further supporting the reliability of the software in residue identification. AUC analysis demonstrated excellent discriminatory performance for all residue severity comparisons, with values between 0.91 and 0.96. For all analyzed comparisons, Fisher’s Exact Test indicated a statistically significant association (*p* < 0.001) between the software classifications and the expert consensus report (Table 2).

**Table 2:** Software performance measures for residues in the pyriform sinuses, considering the consensus report as a reference.

Variables	Accuracy	AUC	Sensitivity	Specificity	Precision	VP+ VP-	RV+ RV-	Fisher’s test
Presence vs. absence of trace	0.95	0.95	0.91	1.00	1.00	1.00 0.91	> 1.00 0.07	0.001*
Presence vs. absence of mild residue	0.95	0.95	1.00	0.91	0.92	0.91 1.00	13 0	0.001*
Mild vs. trace	0.95	0.95	1.00	0.91	0.92	0.91 1.00	13 0	0.001*
Presence vs. absence of moderate residue	0.91	0.91	1.00	0.83	0.85	0.83 1.00	7 0	0.001*
Moderate vs. mild	0.91	0.91	1.00	0.83	0.85	0.83 1.00	7 0	0.001*
Presence vs. absence of severe residue	0.95	0.95	1.00	0.90	0.92	0.90 1.00	13 0	0.001*
Severe vs. mild	0.95	0.95	1.00	0.90	0.92	0.90 1.00	13 0	0.001*
Severe vs. moderate	0.95	0.96	1.00	0.90	0.92	0.90 1.00	13 0	0.001*

*Statistically significant (*p* < 0.05) – Fisher’s Exact Test. AUC = Area Under the Curve; PPV = positive predictive value; NPV = negative predictive value; LR + = positive likelihood ratio; LR− = negative likelihood ratio.

Table 3 summarizes the software’s performance in identifying pyriform sinus residues by YPR-SRS severity. The results indicate consistently high diagnostic performance, with accuracy and AUC values predominantly equal to or close to 1.00 across most comparisons, reflecting excellent overall classification ability. Sensitivity, specificity, and precision were uniformly high, frequently reaching 1.00, demonstrating robust identification of both the presence and absence of residue across severity levels. Across all comparisons, Fisher’s Exact Test revealed a statistically significant association (*p* < 0.001) between the software outputs and the expert consensus report (Table 3).

**Table 3:** Performance measures of the software for vallecular residue, using the consensus report as the reference standard.

Variables	Accuracy	AUC	Sensitivity	Specificity	Precision	VP+ VP-	RV+ RV-	Fisher’s test
Presence vs. absence of trace	0.95	0.95	0.91	1.00	1.00	1.00 0.92	> 1.00 0.07	0.001*
Presence vs. absence of mild residue	1.00	1.00	1.00	1.00	1.00	0.91 1.00	13 0	0.001*
Trace vs. mild residue	0.95	0.95	0.91	1.00	1.00	0.92 1.00	> 1.00 0.08	0.001*
Presence vs. absence of moderate residue	1.00	1.00	1.00	1.00	1.00	1.00 1.00	> 1.00 0	0.001*
Presence vs. absence of severe residue	1.00	1.00	1.00	1.00	1.00	0.83 1.00	7 0	0.001*
Moderate vs. severe	0.91	0.91	0.83	1.00	1.00	1.00 0.85	> 1.00 0.16	0.001*
Presence vs. absence of trace	0.95	0.95	0.91	1.00	1.00	1.00 0.92	> 1.00 0.07	0.001*
Presence vs. absence of mild residue	1.00	1.00	1.00	1.00	1.00	0.91 1.00	13 0	0.001*

*Significant value (*p* < 0.05) – Fisher’s Exact Test. AUC = Area Under the Curve; VP + = positive predictive value; VP - = negative predictive value; RV + = positive likelihood ratio; RV- = negative likelihood ratio.

The agreement between the software and the consensus report, evaluated using Cohen’s Kappa coefficient, was considered almost perfect for the presence of residue in the pyriform sinuses (Kappa = 0.87) and the valleculae (Kappa = 0.91). Figure 5c, d illustrates the distribution of the results.

## Discussion

The framework proposed in this study demonstrated high accuracy in detecting swallowing-related events associated with oropharyngeal dysphagia, based on the analysis of a sample of 60 videos from individuals with a mean age of 64 years, predominantly male, and diagnosed with Parkinson’s disease (PD) as the underlying condition. These results corroborate the literature that highlights the high prevalence of dysphagia in individuals with neurological impairment^1,23,2436]^. Analysis of residues revealed statistically significant results across all severity levels of the YPR-SRS scale, as well as in the identification of penetration and aspiration events, with agreement values classified as almost perfect, according to the kappa criteria proposed by Landis and Koch^22^. These findings suggest that the proposed framework may serve as an assistive system for clinicians in visuoperceptive analysis, providing objective metrics without replacing expert judgment^7,10,11^.

The main methodological contribution of this study, in comparison with previous works^13,16^, lies in the integration of precise anatomical tracking and image optimization aimed at reducing visual artifacts typical of FEES, such as reflections and shadows. This approach enabled a more stable and reproducible quantification of residues in the regions of interest, resulting in greater consistency in the identification of clinically relevant events and performance comparable to^13^ or superior to^16^, based on objective performance metrics commonly reported in studies addressing penetration and aspiration. Additionally, it advances by operationalizing the application of an ordinal residue severity scale, through the computational implementation of previously defined visual criteria, an approach that has not been previously explored in the FEES context, and contributes to improved consistency in the assessment of pharyngeal residues^7^.

The proposed framework aligns with the paradigm of computer-assisted detection, functioning as a clinical decision-support tool that automatically highlights penetration, aspiration, and pharyngeal residue events to guide and structure clinician-led visuoperceptual analysis rather than acting as a screening system or autonomous diagnostic tool.

Given the variability described in the literature regarding the identification of these events in FEES, the analysis of the diagnostic performance of automated systems becomes particularly relevant. Considering that failures in detecting penetration and aspiration may compromise clinical decision-making related to swallowing safety^25^, the reliability of such methods should be examined using metrics that reflect their ability to reproduce consistent clinical decisions, such as sensitivity, specificity, and discriminative capacity^26,27^. In this context, the results indicate that the software was able to reliably identify the presence or absence of penetration and aspiration events based on predefined criteria described in the section Combining AI and Image Processing for Classification of Residues and Identification of Aspiration and Penetration, reproducing decisions consistent with expert consensus^22^.

Regarding the automated classification of residue severity according to the YPR-SR, the literature consistently shows that visual assessment, although widely established in clinical practice, presents limitations related to reproducibility and sensitivity^9,28,29^, which has historically motivated the development of quantitative methods in instrumental examinations, as observed in videofluoroscopy with the proposal of the Normalized Residue Ratio Scale^30^. In the context of FEES, however, previous computational approaches have predominantly focused on event detection, without advancing toward the objective operationalization of clinical residue severity scales^13,16^, which, together with lighting adjustment, constituted a differential of the present work.

In the pyriform sinus region, the use of the automated system was characterized by high accuracy and strong discriminative capacity in identifying and classifying residues across different severity levels^26,27^. This finding underscores the advantage of the automated approach, based on anatomical tracking and lighting adjustment, in an anatomical site where perceptual assessment is particularly sensitive to technical examination factors^31–33^. Nonetheless, a minor limitation was observed in distinguishing between the absence and presence of trace residues, a condition widely reported in the literature, indicating that small amounts of residue in the pyriform sinuses may be confused with secretions or influenced by contrast, opacity, and coating effects during FEES^29,32–35^. Despite this, overall performance remained consistent with the application of the YPR-SRS visual criteria in this anatomical region.

In the valleculae, the framework proposed demonstrated high accuracy in detecting and classifying pharyngeal residues, reliably distinguishing between the different severity levels of the YPR-SRS. The system showed consistent performance across all comparisons, reflecting high sensitivity, specificity, and precision, even in subtle distinctions between severity levels^26,27^. Although the software showed some discrepancies in differentiating between moderate and severe residues, this result is expected, as these categories, due to their high volume, are more susceptible to variations in the distribution and appearance of the material^29,34^. Despite this, the overall discriminative capacity remained high^22,27^, reinforcing the reliability of the framework and its potential to enhance the reproducibility and standardization of residue analysis in FEES^7,10,11^.

This performance can be explained by the fact that both penetration–aspiration events and the severity of pharyngeal residue on FEES are visually defined by the spatial relationship between the bolus and the glottic plane. Therefore, frames corresponding to the optimal visualization of these anatomical landmarks provide a robust and consistent visual basis for identifying false passages and classifying residue according to established criteria. It is important to note that the analysis performed by this system is based on endoscopic visual data and does not take into account physiological factors such as cough effectiveness or bolus clearance, which require professional clinical assessment.

Although two-dimensional imaging represents a simplified projection of complex three-dimensional anatomical structures, this methodological approach is essential for enabling objective quantification and standardization of visual analysis in instrumental swallowing assessments. The YPR-SRS was adopted in this study as a standardized operational visual criterion, as it is a widely used and internationally validated scale developed specifically for the anatomical assessment of pharyngeal residues, with validation in both static two-dimensional images and video analysis, as described in the original study^7,10,11^. However, it should be emphasized that the YPR-SRS is not intended to measure the functional aspects of swallowing but rather to classify residue severity based on defined anatomical criteria^7^. Accordingly, it was used in this study as a consistent criterion for comparative and automated analyses, rather than as an absolute functional standard.

Thus, based on standardized data and objective metrics, the proposed framework functions as a clinical decision-support tool, facilitating the organization and reproducibility of visual analysis and assisting clinicians in correlating FEES findings with patients’ physiological responses, such as cough effectiveness and bolus clearance, which remain under direct clinical judgment. Through objective implementations and the provision of optimal glottic plane visualization for the identification of penetration–aspiration events and YPR-SRS–based classification of pharyngeal residues, the system establishes an anatomically grounded framework that supports visuoperceptual analysis without replacing clinical judgment and may be particularly useful in training contexts involving less experienced clinicians^7–9^.

However, as the study sample and expert reports, developed by consensus and without blinding, were obtained from a single-center database, it is not possible to assert that the results surpass those observed in routine clinical practices. The manual selection of swallowing intervals, use of dyed boluses, equipment variability, and predominance of specific disease populations may limit the generalizability of the findings, as well as the non-inclusion of analyses of underlying pathophysiological mechanisms, particularly in complex residue patterns involving the posterior tracheal wall or deep subglottic regions. Future studies aim to incorporate blinded assessments, fully automated event detection, multicenter and more diverse populations, and analyses of underlying physiopathological mechanisms of swallowing.

## Conclusion

The developed framework demonstrated robust accuracy in detecting penetration, aspiration, and in the automated classification of pharyngeal residues, to our knowledge, one of the first approaches to operationalize classification according to the YPR-SRS. The agreement with experts was considered almost perfect according to the principles of the kappa index, highlighting its assistive potential for FEES analysis and support for clinical decision-making without replacing expert judgment, while also supporting the training of professionals involved in the assessment of oropharyngeal dysphagia. Future studies aim to incorporate blinded assessments, fully automated event detection, multicenter and more diverse populations.

### Limitations

This study has some limitations, including the use of expert diagnoses derived from a single database and established by consensus without blinding, the partial reliance on manual selection of swallowing intervals, and factors limiting generalizability, such as the use of dyed boluses, equipment variability, and the predominance of specific disease populations.

## Data Availability

The datasets used and/or analyzed during the current study are available from the corresponding author on reasonable request.

## Abbreviations

AI: Artificial intelligence
AUC: Area under the curve
CAAE: Certificate of presentation for ethical consideration
CSV: Comma-separated values
DLC: DeepLabCut
FEES: Fiberoptic endoscopic evaluation of swallowing
FPS: Frames per second
HSV: Hue, saturation, value
HUOL: Onofre Lopes University Hospital
IDDSI: International dysphagia diet standardisation initiative
LAB: Lightness, a, b color space
STARD: An updated list of essential items for reporting diagnostic accuracy studies
MAE: Mean absolute error
NLR: Negative likelihood ratio
NPV: Negative predictive value
OD: Oropharyngeal dysphagia
OpenCV: Open source computer vision library
PD: Parkinson’s disease
PLR: Positive likelihood ratio
PPV: Positive predictive value
ResNet-50: Residual neural network with 50 layers
RGB: Red, green, blue
STROBE: Strengthening the reporting of observational studies in epidemiology
UFRN: Federal University of Rio Grande do Norte
VFSS: Videofluoroscopic swallowing study
VP+: Positive predictive value
VP−: Negative predictive value
YPR-SRS: Yale pharyngeal residue severity rating scale
